# Arbuscular Mycorrhiza Improves Photosynthesis and Restores Alteration in Sugar Metabolism in *Triticum aestivum* L. Grown in Arsenic Contaminated Soil

**DOI:** 10.3389/fpls.2021.640379

**Published:** 2021-03-11

**Authors:** Samta Gupta, Sarda Devi Thokchom, Rupam Kapoor

**Affiliations:** Department of Botany, University of Delhi, New Delhi, India

**Keywords:** arbuscular mycorrhizal fungi, arsenic stress, wheat, photosynthesis, sucrose, starch

## Abstract

Contamination of agricultural soil by arsenic (As) is a serious menace to environmental safety and global food security. Symbiotic plant–microbe interaction, such as arbuscular mycorrhiza (AM), is a promising approach to minimize hazards of As contamination in agricultural soil. Even though the potential of AM fungi (AMF) in redeeming As tolerance and improving growth is well recognized, the detailed metabolic and physiological mechanisms behind such beneficial effects are far from being completely unraveled. The present study investigated the ability of an AM fungus, *Rhizophagus intraradices*, in mitigating As-mediated negative effects on photosynthesis and sugar metabolism in wheat (*Triticum aestivum*) subjected to three levels of As, viz., 0, 25, and 50 mg As kg^–1^ of soil, supplied as sodium arsenate. As exposure caused significant decrease in photosynthetic pigments, Hill reaction activity, and gas exchange parameters such as net photosynthetic rate, stomatal conductance, transpiration rate, and intercellular CO_2_ concentration. In addition, As exposure also altered the activities of starch-hydrolyzing, sucrose-synthesizing, and sucrose-degrading enzymes in leaves. Colonization by *R. intraradices* not only promoted plant growth but also restored As-mediated impairments in plant physiology. The symbiosis augmented the concentration of photosynthetic pigments, enhanced Hill reaction activity, and improved leaf gas exchange parameters and water use efficiency of *T. aestivum* even at high dose of 50 mg As kg^–1^ of soil. Furthermore, inoculation with *R. intraradices* also restored As-mediated alteration in sugar metabolism by modulating the activities of starch phosphorylase, α-amylase, β-amylase, acid invertase, sucrose synthase, and sucrose-phosphate synthase in leaves. This ensured improved sugar and starch levels in mycorrhizal plants. Overall, the study advocates the potential of *R. intraradices* in bio-amelioration of As-induced physiological disturbances in wheat plant.

## Introduction

Environmental arsenic (As) contamination is a global agricultural, environmental, and health issue owing to its highly carcinogenic and toxic nature. As is ingressed into the environment, through natural processes, viz., weathering of As-rich rocks, volcanic activity, or anthropogenic activities, namely mining, unwarranted use of As-based pesticides, and irrigation with groundwater contaminated with As in agriculture (Khalid et al., 2017; Abbas et al., 2018). It is a non-essential metalloid and hence not required in any specific metabolic reactions in plants. Both organic and inorganic species of As are present in nature, with the latter being more mobile and toxic than the organic As species. While arsenate [As(V)] exists in oxidized environment, arsenite [As(III)] dominates in reduced environment (Khalid et al., 2017). Being an analog of inorganic phosphate (Pi), As(V) is transported across plasma membrane through the phosphate transport systems. Thus, it competes and interferes with Pi uptake and metabolism (Stoeva and Bineva, 2003) in plants. Once taken up by the plants, various Pi transporters enable easy movement of As(V) from one cellular compartment to another (Finnegan and Chen, 2012). In doing so, all parts of cellular metabolism get exposed to the toxicant. For instance, it replaces phosphate in ATP and forms an unstable ADP–As complex, thereby leading to interference of energy flow in cells (Meharg, 1994).

As can affect the growth and productivity of plants due to a surfeit of morphological, physiological, and biochemical alterations (Chandrakar et al., 2016; Srivastava et al., 2017). Production of reactive oxygen species (ROS) is one of the most perilous biochemical effects of As at the subcellular level, causing non-repairable damage to various macromolecules, such as lipids, proteins, carbohydrate, and DNA (Talukdar, 2013; Chandrakar et al., 2016).

As is reported to disrupt net photosynthetic rate (Pn) in plants (Gusman et al., 2013), due to disturbances either in the photochemical or biochemical steps or both. Light-harvesting apparatus of plants gets affected by As via reduction of chlorophyll (Chl) concentrations and photosynthetic activity (Anjum et al., 2011; Emamverdian et al., 2015). Rate of CO_2_ fixation and activity of photosystem (PS) II also get reduced considerably under As exposure (Stoeva and Bineva, 2003). As also negatively influences photochemical efficiency and heat dissipation capacity, thereby upholding changes in gas exchange rate and fluorescence emission (Chandrakar et al., 2016; Debona et al., 2017). These toxic effects of As on photosynthetic parameters of plants are manifested in the forms of diminution in growth, wilting, and violet color development of leaves (Musil et al., 2014).

In addition to disturbing Pn in plants, As has also been demonstrated to influence carbon partitioning and sugar metabolism in plants (Jha and Dubey, 2004; Choudhury et al., 2010; Sil et al., 2019; Majumder et al., 2020). Metabolism of basic carbohydrates such as sugars and starch in plants is deleteriously affected under As stress (Chandrakar et al., 2016). Sucrose and starch, the resultant products of photosynthesis, act as regulators of stress responses and play a principal role in gene expression under abiotic stresses (Rosa et al., 2009). Accumulation of soluble sugars can take place in response to the stress (Gramss, 2012) as a means to cope up with As-mediated oxidative stress with a discrepancy in the contents of reducing sugars (RSs) and non-RSs (NRSs) (Jha and Dubey, 2004). Conversion of NRSs, primarily sucrose, into RSs (hexoses) is generally observed under As stress, indicating the suppression of sucrose synthesis (Jha and Dubey, 2004). Inhibition of starch-degrading enzymes’ activities as a consequence of As-mediated plant toxicity has also been reported (Jha and Dubey, 2004). Considering the aspect that plant directly exposed to As require more energy and carbon molecules to cope with the stress, studies focusing on photosynthesis and sugar metabolism under As stress could help in developing relevant strategies to confer plant tolerance toward As-instigated toxicity.

Among the cereals, wheat (*Triticum aestivum* L.) is the second most important crop consumed mainly as a source of carbohydrate as well as dietary protein and minerals. It ranks first in terms of global consumption (Food and Agriculture Organization (FAO), 2020). The major concern arising in wheat cultivation is the concurrence of As-contamination area with its cultivated area. For instance, in India, wheat is cultivated under six diverse agro-climatic zones, wherein Indo-Gangetic Plains comprising the two zones form major wheat-cultivating plain (Rasheed et al., 2018). Wheat grains harvested from this region also report high As accumulation resulting from unparalleled As biomagnification. The risks posed by the contaminant from wheat grains, however, do not outweigh the global demand of wheat and its products. Thus, with the increasing global population, production of wheat needs to meet the global demand as well as reduce the toxic content in its nutritional composition. In this context, use of arbuscular mycorrhizal fungi (AMF) in agriculture has been reported to sustainably improve plant’s tolerance to various heavy metal stresses (Sharma et al., 2017; Zhan et al., 2018; Alam et al., 2019; Wu et al., 2020).

Arbuscular mycorrhizal fungi, belonging to the subphylum Glomeromycotina, are the most widespread root symbiotic fungi, reportedly developing mutualistic associations with approximately 80% of terrestrial plants (Smith and Read, 2008). It was reported that colonization by AMF not only improves growth and biomass of wheat plants but also aids the host to surmount As-induced P deficiency and also maintains higher P/As ratio when compared with non-colonized plants (Sharma et al., 2017). In previous study, it was found that AMF colonization can reduce uptake of As and its translocation to wheat grains. Altered mineral status and photosynthetic parameters mediated by AMF in stressed plants as mitigation strategy is also accountable to affect carbohydrate metabolism in plants. In addition, carbon handling is a fundamental aspect of plant–AMF interaction, as a significant fraction of the plant’s photosynthates (sugars) is directed toward AMF (Bago et al., 2000) and thus can alter carbohydrate metabolism. In line with this, studies on the effect of As contamination and AMF inoculation on physiological processes such as photosynthesis and carbohydrate metabolism are restricted to assessment at seedling stage (Jha and Dubey, 2004; Choudhury et al., 2010; Sil et al., 2019). Keeping this in mind, the present study was performed to assess the ability of *Rhizophagus intraradices* to (i) promote photosynthesis-related activities (pigment concentration, Hill reaction activity, gas exchange parameters, and Chl *a* fluorescence) and (ii) stimulate the activities of enzymes involved in sucrose and starch metabolism, in wheat plants exposed to three levels of As.

## Materials and Methods

### Plant Material and Fungal Inoculum

Seeds of wheat variety HD-2967 were procured from Agricultural Technology Information Centre, Indian Agricultural Research Institute (IARI), New Delhi, India. *R. intraradices* (accession number CMCCWep319) inoculum was provided by Center for Mycorrhizal Culture Collection, The Energy and Resources Institute, New Delhi, India. Proliferation of fungal spores was carried out in sterile soil using *Sorghum bicolor* L. as trap plant (Kapoor et al., 2002). After confirmation of colonization by mycorrhizal fungi (Phillips and Hayman, 1970), the plants were let to dry progressively to stimulate spore formation under shade (INVAM, 2014). The inoculum prepared consisted of finely chopped *R. intraradices* colonized roots and dried soil containing approximately 120–150 spores 10 g^–1^ of soil.

### Experimental Layout

A pot-based (3 kg soil per pot) experiment was set up in the Botanical Garden, Department of Botany, University of Delhi. Three As levels (0, 25, and 50 mg As kg^–1^ of soil) and two mycorrhizal treatments [inoculated with *R. intraradices* (M) and non-mycorrhizal (NM) ones] were the factors under consideration. In total, there were six treatments structured in complete randomized block design.

### Soil Treatment and Plant Growth Conditions

Soil was collected from the Botanical Garden (0–15 cm in depth) of the Department of Botany, University of Delhi, and air-dried for use for the experiment. The soil was sieved through 2-mm sieve and was maintained in a 3:1 (v:v) ratio by mixing thoroughly with sand (henceforth termed as soil). Analysis of available nutrients in soil was done prior to As addition at the Division of Soil Science and Agricultural Chemistry, IARI, New Delhi, India. The soil contained adequate levels of N (2,144.82 mg kg^–1^ of soil), P (1,868.74 mg kg^–1^ of soil), K (5,703.82 mg kg^–1^ of soil), Ca (268.27 mg kg^–1^ of soil), and Mg (103.56 mg kg^–1^ of soil). The soil was autoclaved at 121°C and 15 psi for 1 h, twice, to eradicate any existing microbes, followed by treatment with three As concentrations, namely, 0, 25, and 50 mg As kg^–1^ of soil, prepared using sodium arsenate (Na_2_HAsO_4_.7H_2_O). Selection of these As concentrations was based on a previous study done in wheat (Sharma et al., 2017) and concentrations of As reported in agricultural soils of Southeast Asia (Alam and Sattar, 2000; Rahman et al., 2013; Tong et al., 2014; Shrivastava et al., 2017). These soil treatments are hereafter referred to as 0, 25, and 50As. The required quantity of Na_2_HAsO_4_.7H_2_O for the three different As levels was dissolved in distilled water (50 ml) and then mixed with soil thoroughly in plastic trays to ensure homogenous As distribution. The soil was left in trays to equilibrate with recurrent cycles of saturation and air-drying for a period of 1 month and dispensed in pots (Cox and Kovar, 2001). Air-dried soil (100 mg) of each treatment was microwave digested using HNO_3_ and H_2_O_2_. Volume of the digest was made up to 40 ml with Milli-Q water and later filtered using 0.2-μm membrane filter and analyzed for total As, using inductively coupled plasma mass spectrometer (ICP-MS 7900, Agilent Technologies, Japan). The total As in soil of 0, 25, and 50As was 15.2 μg kg^–1^, 23.6 mg kg^–1^, and 48.9 mg kg^–1^, respectively.

Ten surface-sterilized (using 1% sodium hypochlorite solution) wheat seeds were sown per pot. Mycorrhizal plants were provided with 20 g of *R. intraradices* inoculum dispensed at a depth of 2 cm in each pot. NM plants were supplied with 20 ml of soil washing of an equal amount of soil filtered through Whatman No. 1. This guaranteed introduction of microbial populations other than any other propagules, along with an equal amount of autoclaved soil mix to exclude any other variables. Pots were placed outside in open ground at the Botanical Garden, Department of Botany, University of Delhi, under natural conditions (rabi season; 9–16°C), humidity (56–90%), and natural light. Plants were watered to 60% field capacity of soil to avoid drainage of As.

### Plant Sampling

Just before the initiation of florets (i.e., 42 days after sowing), six plants from each treatment were harvested along with roots. The plants were washed with water to remove adhering soil particles. Three plants from each treatment were oven dried at 60°C till a constant weight was recorded. The remaining three were used for determining AMF colonization in roots and biochemical estimation in leaves.

### Mycorrhizal Colonization and Metal Tolerance Index

Colonization of root cortex by *R. intraradices* was confirmed after clearing and staining roots with 5% KOH and 0.05% trypan blue in lactoglycerol following the modified protocol of Phillips and Hayman (1970). Percent root colonization was calculated following the gridline intersect method (Giovannetti and Mosse, 1980). For this, one hundred 1 cm root segments were placed in petri dish with gridlines having 1 cm^2^ boxes. Roots were observed under stereoscope, and horizontal and vertical intersects having mycorrhizal structures were counted. Root colonization was calculated as per the following formula:

$$\mathrm{Mycorrhizal}\mathrm{colonization}\left(\%\right)=\frac{\mathrm{No}.\mathrm{of}\,\mathrm{roots}\,\mathrm{colonized}}{\mathrm{Total}\,\mathrm{no}.\mathrm{of}\,\mathrm{roots}}\times 100$$

Metal tolerance index (MTI) of wheat to As in soil was determined according to Rabie (2005) using the following formula:

$$\mathrm{MTI}\left(\%\right)=\frac{\mathrm{Plant}\,{\mathrm{DW}}_{\left(\mathrm{at}\,\mathrm{particular}\,\mathrm{level}\,\mathrm{of}\,\mathrm{As}\right)}}{\mathrm{Plant}\,{\mathrm{DW}}_{\left(\mathrm{in}\,\mathrm{control}\,\mathrm{soil}\right)}}\times 100$$

where plant DW indicates dry weight of plant (roots and shoots) determined after 42 days of sowing.

### Concentration of As, Mg, P, and N

Oven-dried leaves were analyzed for their nutrient composition. Leaves (100 mg) were finely ground and sieved through a 0.5-mm sieve. Sieved samples were then subjected to acid digestion in a microwave reaction system (Anton Paar make, model: Multiwave PRO) using concentrated nitric acid. The internal temperature limit of the digester was maintained at 200°C for 30 min. The volume of the digest was made to 40 ml with Milli-Q water and later filtered using 0.2-μm membrane filter and analyzed for the estimation of As and other nutrient concentrations. As and Mg concentrations were quantified using ICP-MS (ICP-MS 7900, Agilent Technologies, Japan). While a concentration of P was quantified following the ammonium molybdate blue method described by Allen (1989), N concentration was measured by CHNS analyzer (Elementar Analysis System, Vario Micro Cube, Germany).

### Concentration of Total Protein

The trichloroacetic acid (TCA)–acetone method described by Jia et al. (2019) was used for extraction of total protein. The leaves (100 mg) were ground in liquid nitrogen with pestle and mortar. Five-time volume of TCA/acetone (1:9) was added to the homogenized powder and mixed using a vortex. The mixture was incubated at –20°C for 4 h, followed by centrifugation at 6,000 *g* for 40 min at 4°C. The supernatant was discarded, and the resultant pellet was washed three times with chilled acetone. The precipitate was air-dried and reconstituted in buffer (1:30, v/v) containing sodium dodecyl sulfate (4%), dithiothreitol (DTT; 100 mM) and Tris–HCl (150 mM; pH 8). The mixture was sonicated (80 W for 10 s, intermittent for 15 s) for 10 cycles and boiled for 5 min. After that, the lysate was re-centrifuged for 40 min at 14,000 *g*. The resulting supernatant was filtered with 0.22-μm filter, and total protein was estimated following the Bradford (1976) assay using bovine albumin serum as standard.

### Photosynthesis

#### Photosynthetic Pigments

Intact leaf tissues (100 mg) were dipped in 7 ml of dimethyl sulfoxide and heated at 65°C for 30 min to extract photosynthetic pigments (Hiscox and Israelstam, 1979). The extract was then transferred to a graduated tube. With the use of dimethyl sulfoxide, the final volume of the extract was made up to 10 ml. Absorbance of the extract was read at 453, 645, and 663 nm. Concentration of total Chl (T-Chl), Chl *a*, Chl *b*, and total carotenoids was calculated using the formula of Arnon (1949).

#### Hill Reaction Activity

Hill reaction activity was assayed following the protocol of Vishniac (1957). Leaf samples (1 g) were homogenized in 5 ml of sucrose-phosphate buffer (0.5 M of sucrose in 0.05 M of sodium phosphate buffer; pH 6.2). The homogenate was then centrifuged at 1,000 *g* at 4°C for 10 min. The supernatants collected were re-centrifuged for 15 min at 5,000 *g* at 4°C. Suspensions of chloroplast were made by dissolving the pellets after centrifugation in 5 ml of sucrose-phosphate buffer; 1 ml of chloroplast suspension was then mixed with 4 ml of sucrose-phosphate buffer and 0.5 ml 2,6-dichlorophenolindophenol (DCPIP) (0.03%). Following this, the reaction sets were kept under bright light (irradiance 800–1,000 μmol m^–2^ s^–1^) for 30 min after taking initial absorbance at 610 nm. After discoloration of the reaction mixture, absorbance was again recorded. The differences in the absorbances were estimated, and Hill reaction activity was calculated referring to a standard curve prepared with DCPIP and was expressed as μg DCPIP reduced mg^–1^ Chl min^–1^.

#### Gas Exchange Parameters

Leaf gas exchange parameters such as leaf Pn, transpiration rate (E), stomatal conductance (Gs), and intercellular CO_2_ concentration (Ci) were analyzed on fully expanded young leaves between 9:00 and 11:00 am on a clear sunny day using a portable infrared gas analyzer (Gas Exchange Fluorescence System, GFS-3000). The analyzer was adjusted for leaf surface area (3.00 cm^2^), ambient CO_2_ concentration (ca) (398 ppm), photosynthetic photon flux density (1,000 μmol m^–2^ s^–1^), impeller at 7, and relative humidity inside the cuvette maintained at 35%. Water use efficiency (WUE) was calculated by dividing the value of Pn by E.

#### Chlorophyll *a* Fluorescence

Chl *a* fluorescence was also monitored in terms of minimal fluorescence (Fo), potential efficiency of PSII (Fv/Fo), maximum efficiency of PSII (Fv/Fm), and photochemical quenching coefficient (qP), where Fv is variable fluorescence (Fm – Fo), Fo is minimal fluorescence, and Fm is maximal fluorescence on the adaxial leaf surface, using a portable infrared gas analyzer (Gas Exchange Fluorescence System, GFS-3000). For this, leaf was dark adapted for 30 min and later irradiated by a saturating pulse of 2,000 μmol m^–2^ s^–1^, sufficient for complete oxidation of the reaction centers.

### Sugar Metabolism

#### Total Soluble Sugar

Concentration of total soluble sugar (TSS) was quantified following the phenol sulfuric acid reagent method (Dubois et al., 1956). Samples were homogenized using 80% ethanol. The extracts were then centrifuged for 20 min at 2,000 rpm. Reaction mixture contained 1 ml of supernatant, 0.05 ml of phenol (5%), and sulfuric acid (98%). The mixtures were then incubated in water bath for 20 min at 30°C. Absorbance was measured at 490 nm. With the use of standard curve of glucose, content of TSS was quantified and expressed as mg g^–1^ fresh weight (FW).

#### Reducing Sugar, Non-reducing Sugar, and Reducing Sugar/Non-reducing Sugar Ratio

Concentration of RS was measured following the protocol of Miller (1972). Plant samples (1 g) were extracted in 5 ml of 80% ethanol and later centrifuged at 2,000*g* for 20 min. The collected supernatants were then mixed with 1% 3,5-dinitrosalicylic acid reagent (0.5 ml) followed by incubation for 5 min in boiling water bath. Absorbance of the mixture was read at 515 nm. Concentration of RS was estimated from a standard curve of glucose and expressed as mg g^–1^ FW. Quantification of NRS was done by subtracting the values of RS from that of TSS and expressed as mg g^–1^ FW.

#### Sucrose-Phosphate Synthase Activity

Sucrose-phosphate synthase (SPS) activity was assessed according to Miron and Schaffer (1991). Plant enzyme extract was prepared using the extraction buffer that contained HEPES–NaOH buffer (50 mM; pH 7.5) containing MgCl_2_ (5 mM), EDTA (1 mM), DTT (2.5 mM), and Triton X-100 (0.05%; v/v). The extracts were then centrifuged at 4°C at 10,000 rpm for 10 min. The reaction mixture consisted of HEPES–NaOH buffer (50 mM; pH 7.5), MgCl_2_ (15 mM), fructose-6-phosphate (25 mM), glucose-6-phosphate (25 mM), UDP-glucose (25 mM), and enzyme extract. The mixtures were then incubated at 37°C for 30 min. Termination of the reaction was brought by addition of 30% KOH. Sucrose formed during SPS catalyzed reaction was then estimated, and its activity was expressed as nmol sucrose formed mg^–1^ protein min^–1^.

#### Sucrose Synthase Activity

Activity of SS was estimated following the protocol of Miron and Schaffer (1991). Plant enzyme extract was prepared using HEPES–NaOH buffer (50 mM; pH 7.5) containing MgCl_2_ (5 mM), EDTA (1 mM), DTT (2.5 mM), and Triton X-100 (0.05%; v/v). The obtained extract was then centrifuged at 4°C at 10,000 rpm for 10 min. Reaction mixture included HEPES–NaOH buffer (50 mM; pH 7.5), MgCl_2_ (15 mM), fructose (25 mM), UDP-glucose (25 mM), and enzyme extract. The mixture was then incubated at 37°C for 30 min. The reaction was terminated by adding 30% KOH. Sucrose hydrolyzed during SS catalyzed reaction was estimated, and the enzyme activity was expressed as μmol sucrose hydrolyzed mg^–1^ protein min^–1^.

#### Acid Invertase Activity

Activity of acid invertase (AI) was estimated following the method of Borkowska and Szczerha (1991). Plant samples were homogenized in sodium acetate buffer (10 mM; pH 4.6) containing MgCl_2_ (3.3 mM), EDTA (1 mM), and phenylmethylsulfonyl fluoride (PMSF) (1 mM). Homogenates were then centrifuged for 20 min at 10,000 rpm at 4°C. Assay mixture consisted of sodium acetate buffer (10 mM; pH 4.6), sucrose (0.4 M), and the enzyme extract. The final volume of 1.0 ml was made up. After incubation for 30 min at 30°C, termination of the reaction was brought by addition of Na_2_HPO_4_ (0.5 M). Resulting RSs were then estimated by Nelson–Somogyi method (Nelson, 1944), and activity was expressed as μmol sucrose hydrolyzed mg^–1^ protein min^–1^.

### Starch Metabolism

#### Starch Concentration

Starch concentration was estimated following the protocol of McCready et al. (1950). Residual mass collected after centrifugation (for TSS extraction) was dissolved in distilled water. Later, perchloric acid was added and stirred, followed by centrifugation of the mixture for 20 min at 2,000 rpm. Supernatants were collected and then poured in conical flasks. The total volume was later made up to 100 ml with the addition of distilled water. Starch concentration was measured in 1 ml of filtrate following the same procedure as that of TSS. Starch quantity was then estimated in terms of glucose, and the factor 0.9 was used to convert the values of glucose to starch. Starch concentration was expressed in mg g^–1^ FW.

#### α-Amylase and β-Amylase Activities

Following the method of Bush et al. (1989), the activity of α-amylase was determined. Plant samples were homogenized in sodium acetate buffer (0.1 M; pH 4.8) containing cysteine (5 μM) and centrifuged for at 10,000 rpm 15 min at 4°C. The obtained supernatants were then heated at 70°C for 5 min in the presence of CaCl_2_ (3 mM). Reaction mixture consisted of sodium acetate buffer (0.1 M; pH 4.8), soluble starch (1%) in NaCl (0.15 M), and the enzyme extract. The final volume of the reaction mixture was made up to 4 ml and left for incubation at 30°C for 5 min. Termination of reaction was brought by addition of HCl (6 M). Aliquots (1 ml) were then transferred to conical flasks in which 0.5 ml of IKI solution (0.2% I_2_ in 2% KI) was later added. The final volume was then made up to 25 ml with distilled water. Absorbance was read at 660 nm. Activity of enzyme was expressed as μg of starch hydrolyzed mg^–1^ protein min^–1^.

Activity of β-amylase was estimated following the protocol of Bernfeld et al. (1965). The enzyme was extracted from plant samples in phosphate buffer (pH 7.0) that contained NaCl (0.5 M). Starch solution (1 ml) and properly diluted enzyme (1 ml) were pipetted out and incubated at 25°C for 15 min. The reaction was later stopped by adding 2 ml of 3,5-dinitrosalicylic acid reagent. The reaction mixture was then heated in a water bath (60°C) for 5 min. While the tubes were warm, 1 ml of potassium sodium tartrate solution was added, followed by cooling of the mixture under running tap water. The final volume was made up to 10 ml using distilled water. The absorbance was recorded at 570 nm. Activity of the enzyme was expressed as μg of maltose hydrolyzed mg^–1^ protein min^–1^.

#### Starch Phosphorylase Activity

The activity of starch phosphorylase (SP) was determined following the protocol of Dubey and Singh (1999). Plant samples were homogenized in 50 mM of citrate buffer containing EDTA (1 mM; pH 6.0), β-mercaptoethanol (5 mM), and PMSF (1 mM). Homogenized samples were then centrifuged for 20 min at 10,000 rpm at 4°C. Assay mixture was prepared containing citrate buffer (50 mM), soluble starch (5%; w/v), glucose-1-phosphate (0.1 mM), and enzyme extract; and the total volume was made up to 4.0 ml. With the addition of 5% TCA, the reaction was stopped after 10 min. Reaction mixture was then centrifuged, and the phosphorus content in the supernatant was determined following the method of Fiske and Subbarow (1925). Enzyme activity was calculated as nmol of Pi liberated mg^–1^ protein min^–1^.

### Statistical Analysis

Statistical Package for the Social Sciences Statistics software version 21.0 (SPSS Inc., IBM Corporation, Armonk, NY, United States) was used to analyze the results. Multivariate analysis of variance (MANOVA) was used to evaluate statistical differences among treatments. One-way analysis of variance (ANOVA) was done for comparing the differences between individual means using Tukey’s honestly significant difference (HSD) *post hoc* test. All the values were represented as means of three biological replicates ± standard deviation (SD).

## Results

### Root Colonization by *Rhizophagus intraradices*

Histochemical staining of the roots showed successful colonization of *Triticum aestivum* roots by *R. intraradices*. The presence of As in soil increased per cent colonization. However, the extent of increase varied in the two As concentrations. In comparison with 0As, root colonization increased by 10.1 and 4.2% in 25 and 50As, respectively.

### Metal Tolerance Index and Concentrations of As, Mg, P, N, and Total Protein

As level, mycorrhizal status, and their interaction significantly affected MTI of wheat plants (Table 1). A significant (*p* ≤ *0.05*) reduction in MTI was observed in response to As amendments in soil. In NM plants, an increase in As level from 25 to 50 mg resulted in a decline of MTI by 38.9%. However, plants colonized by *R. intraradices* increased MTI by 91.7% at 25As and 131.5% at 50As when compared with their corresponding NM plants (Table 1).

**TABLE 1:** Arbuscular mycorrhizal colonization; MTI; and concentrations of As, P, Mg, N, and total proteins in leaves of *Triticum aestivum* in response to *Rhizophagus intraradices* inoculation (M, mycorrhizal; NM, non-mycorrhizal) and As addition to the soil.

As level (mg As kg^−1^ soil)	AMF status	Mycorrhizal colonization (%)	MTI (%)	As (μg g^–1^ DW)	P (mg g^–1^ DW)	Mg (mg g^–1^ DW)	N (mg g^–1^ DW)	Total protein (mg g^–1^ FW)
0	NM		–	0.48 ± 0.01e	3.24 ± 0.08b	5.46 ± 0.002d	44.56 ± 0.98b	112.54 ± 1.45b
	M	56.00 ± 1.00c	–	0.04 ± 0.01f	5.77 ± 0.14a	6.95 ± 0.003a	52.63 ± 0.97a	119.99 ± 2.56a
25	NM	–	59.82 ± 6.95c	12.54 ± 1.36c	2.15 ± 0.12d	5.05 ± 0.004e	38.46 ± 0.61d	80.72 ± 1.06e
	M	61.66 ± 1.53a	114.67 ± 10.35a	7.12 ± 1.05d	2.81 ± 0.07c	6.70 ± 0.10b	41.30 ± 0.91c	92.78 ± 2.50c
50	NM	–	36.50 ± 4.95d	24.35 ± 0.77a	1.48 ± 0.09e	4.37 ± 0.001f	27.76 ± 1.15f	67.72 ± 2.93f
	M	58.33 ± 0.58b	84.49 ± 5.48b	14.07 ± 0.11b	2.78 ± 0.05c	6.01 ± 0.004c	34.53 ± 0.60e	88.50 ± 1.96d
Significance As		***	*	***	***	***	***	***
AMF		–	***	***	***	***	***	***
As × AMF		–	***	*	***	***	***	***

Values represent means of three biological replicates ± SD. Different letters within the column represent significant difference among the treatments at *p* ≤ 0.05, derived from Tukey HSD.

* and *** represent significance at *p* ≤ 0.05 and *p* ≤ 0.001, respectively, derived from two-way ANOVA.

*As, arsenic; AMF, arbuscular mycorrhizal fungi; Mg, magnesium; MTI, metal tolerance index; N, nitrogen; P, phosphorus; HSD, honestly significant difference.*

Two-way ANOVA showed that As additions in soil and *R. intraradices* inoculation independently as well as interactively affected concentration of As in wheat leaves (Table 1). With increase in As level in soil, there was a concomitant increase in leaf As concentration as substantiated by a 94.1% increase in plants of 50As when compared with that of 25As. Nevertheless, colonization by *R. intraradices* decreased leaf As concentrations at all As levels with respect to their corresponding NM plants.

Concentration of P declined in wheat leaves in response to presence of As in soil (Table 1). M plants possessed higher P concentration in leaves over NM plants at all As levels. When compared with NM plants, P concentrations increased by 78.1, 30.6, and 87.8% at 0, 25, and 50As, respectively, in M plants. A similar effect was observed on concentrations of other nutrients and total protein. Concentrations of Mg, N, and total protein declined in wheat leaves in response to As stress (Table 1). Colonization by AMF improved the antagonistic effect inflicted by As and increased their concentration significantly when compared with their NM counterparts at all As levels. At 50As, their concentrations were adversely affected, and the ameliorative effect of *R. intraradices* was evident with 37.5, 24.4, and 30.7% increase in Mg, N, and total protein concentrations, respectively.

### Photosynthesis

#### Photosynthetic Pigments

Presence of As in soil, mycorrhizal status, and interaction of both the factors had significant influence on T-Chl, Chl *a*, Chl *b*, total carotenoids, and Chl *a*/*b* ratio (Table 2). With an increase in As level in soil, leaves of wheat plants showed decline in concentration of T-Chl, Chl *a*, Chl *b*, and total carotenoids. When compared with 0As, plants of 25As treatment showed decline of 34.1, 29.6, 50.0, and 27.4% in concentrations of T-Chl, Chl *a*, Chl *b*, and total carotenoid, respectively. A similar but more severe effect was observed on photosynthetic pigments at high As level. Plants grown at 50As showed 56.1, 49.0, 80.0, and 38.35% decline in concentrations of T-Chl, Chl *a*, Chl *b*, and total carotenoid, respectively, over plants grown at 0As. Colonization by *R. intraradices* augmented the concentrations of these photosynthetic pigments at all As levels (Table 2). Chl *a*/*b* ratio also increased with increasing As concentration in soil in NM plants. In M plants, the ratio was significantly (*p* ≤ *0.05*) lower than that of NM plants at 25 and 50As. In response to mycorrhizal colonization, the concentration of T-Chl increased by 12.2, 40.7, and 83.3% at 0, 25, and 50As, respectively. Similarly, total carotenoid concentration at the abovementioned As levels increased by 12.3, 26.4, and 33.3%, respectively, in M plants over NM plants.

**TABLE 2:** Concentrations of total T-Chl, Chl *a*, Chl *b*, total carotenoids, and Chl *a/b* ratio in leaves of *Triticum aestivum* in response to *Rhizophagus intraradices* inoculation (M, mycorrhizal; NM, non-mycorrhizal) and As addition to soil.

As level (mg As kg^−1^ soil)	AMF status	T-Chl (mg g^–1^ FW)	Chl *a* (mg g^–1^ FW)	Chl *b* (mg g^–1^ FW)	Chl *a*/*b* ratio	Total carotenoids (mg g^–1^ FW)
0	NM	2.05 ± 0.04b	1.55 ± 0.07a,b	0.50 ± 0.02a	3.09 ± 0.31c	0.73 ± 0.06a
	M	2.30 ± 0.05a	1.72 ± 0.09a	0.58 ± 0.04a	2.98 ± 0.40c	0.82 ± 0.09a
25	NM	1.35 ± 0.06c	1.09 ± 0.06c	0.25 ± 0.01c	4.29 ± 0.34b	0.53 ± 0.03d
	M	1.90 ± 0.17b	1.51 ± 0.02b	0.39 ± 0.006b	3.83 ± 0.12b,c	0.67 ± 0.07b
50	NM	0.90 ± 0.09d	0.79 ± 0.08d	0.10 ± 0.01d	7.32 ± 0.36a	0.45 ± 0.05e
	M	1.65 ± 0.11c	1.26 ± 0.04c	0.38 ± 0.07b	3.31 ± 0.54b,c	0.60 ± 0.02c
Significance						
As		***	***	***	***	***
AMF		***	***	***	***	**
As × AMF		***	**	***	***	***

Values represent means of three biological replicates ± SD. Different letters within the column represent significant difference among the treatments at *p* ≤ 0.05, derived from Tukey HSD.

** and *** represent significance at *p* ≤ 0.01 and *p* ≤ 0.001, respectively, derived from two-way ANOVA.

*As, arsenic; AMF, arbuscular mycorrhizal fungus; T-Chl, total chlorophyll; Chl *a*, chlorophyll *a*; Chl *b*, chlorophyll *b*; HSD, honestly significant difference.*

#### Hill Reaction Activity

**FIGURE 1:**
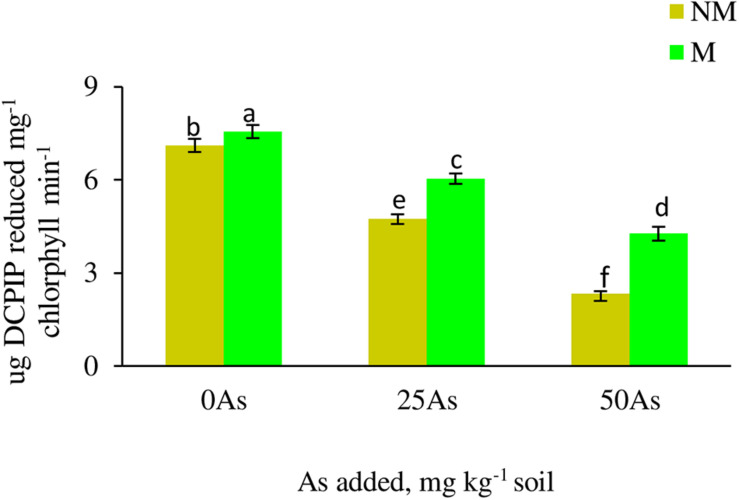
Hill reaction activity in leaves of *Triticum aestivum* in response to *Rhizophagus intraradices* inoculation (M, mycorrhizal; NM, non-mycorrhizal) and As addition to the soil. Values represent means of three biological replicates ± SD. Different letters represent significant difference at *p* ≤ *0.05*, derived from Tukey’s honestly significant difference (HSD). 0As, 0 mg As kg^–1^ soil treatment; 25As, 25 mg As kg^–1^ soil treatment; 50As, 50 mg As kg^–1^ soil treatment.

Hill reaction activity of plant significantly (*p* ≤ *0.05*) decreased with increased As concentration in soil (Figure 1). Colonization by *R. intraradices* showed increase in the activity at all As levels when compared with NM plants, with a maximum of 40% increase reported at 50As.

#### Gaseous Exchange

**FIGURE 2:**
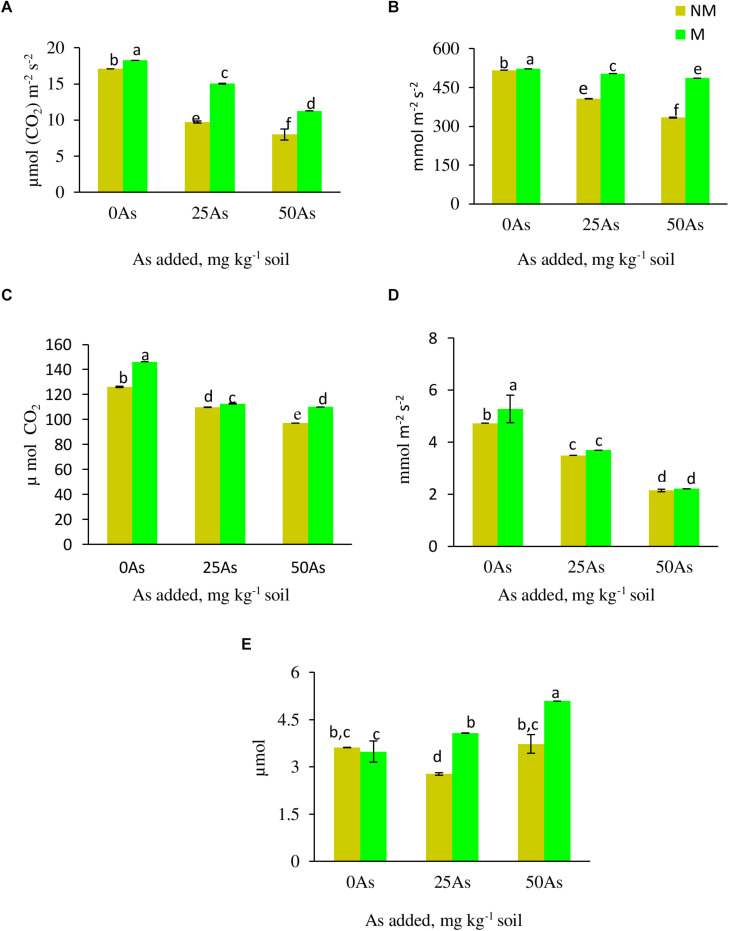
Gas exchange parameters **(A)** net photosynthetic rate, Pn; **(B)** stomatal conductance, Gs; **(C)** intercellular CO_2_ concentration, C_*i*_; **(D)** transpiration rate, E; and **(E)** water use efficiency, WUE, of *Triticum aestivum* in response to *Rhizophagus intraradices* inoculation (M, mycorrhizal; NM, non-mycorrhizal) and As addition to the soil. Values represent means of three biological replicates ± SD. Different letters represent significant difference at *p* ≤ *0.05*, derived from Tukey’s honestly significant difference (HSD). 0As, 0 mg As kg^–1^ soil treatment; 25As, 25 mg As kg^–1^ soil treatment; 50As, 50 mg As kg^–1^ soil treatment.

All the gas exchange parameters, except WUE, showed decline with corresponding increase in As concentration in soil (Figure 2). Mycorrhizal colonization assisted wheat plants in maintaining better gaseous exchange when compared with NM plants. At 0As, M plants significantly increased Pn and Ci by 6.0 and 15.9%, respectively, over their NM counterparts. The influence of *R. intraradices* in improving gaseous exchange was more evident when plants were exposed to low As stress, wherein M plants maintained 55.1, 24.1, 5.7, 2.6, and 46.9% higher Pn, Gs, E, Ci, and WUE, respectively, when compared with NM plants. Likewise, at high As level, the abovementioned parameters increased by 40.9, 45.7, 3.2, 13.3, and 36.5%, respectively, in M plants over their NM counterparts.

#### Chlorophyll *a* Fluorescence

**FIGURE 3:**
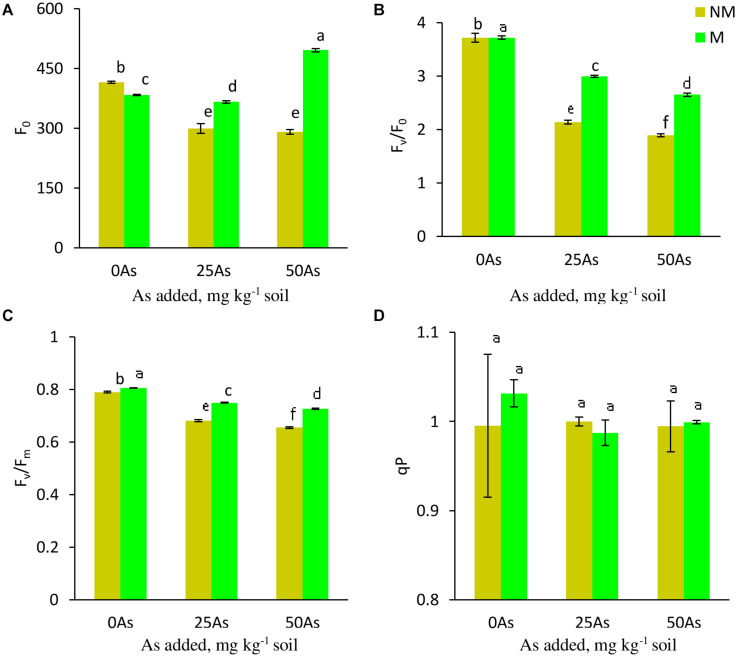
Chl *a* fluorescence **(A)** minimal fluorescence, Fo; **(B)** potential efficiency of PSII, Fv/Fo; **(C)** maximum efficiency of PSII, Fv/Fm; and **(D)** photochemical quenching coefficient, qP, in leaves of *Triticum aestivum* in response to *Rhizophagus intraradices* inoculation (M, mycorrhizal; NM, non-mycorrhizal) and As addition to the soil. Values represent means of three biological replicates ± SD. Different letters represent significant difference at *p* ≤ *0.05*, derived from Tukey’s honestly significant difference (HSD). 0As, 0 mg As kg^–1^ soil treatment; 25As, 25 mg As kg^–1^ soil treatment; 50As, 50 mg As kg^–1^ soil treatment.

While As level, mycorrhizal status, and their interaction showed a significant (*p* ≤ *0.001*) effect on Fo, Fv/Fo, and Fv/Fm, a non-significant effect was reported on qP (Table 3). Exposure to As stress led to decrease of Fo, Fv/Fo, and Fv/Fm in a dose-dependent manner (Figure 3). Colonization by *R. intraradices* led to an overall increase in Fv/Fm and Fv/Fm as compared with NM plants, although the extent of increase varied with each As level. Contrary to this, *R. intraradices* colonization decreased Fo at 0As by 7.7%; however, it increased by 22.3 and 70.1% at 25 and 50As, respectively, when compared with NM plants.

**TABLE 3:** Two-way analysis of variance showing the effect of As level, mycorrhizal status, and their interaction on various physiological attributes of *Triticum aestivum*.

Parameter	As level	AMF status	As × AMF
Hill reaction activity	***	***	***
Pn	***	***	***
E	***	*	ns
Gs	***	***	***
Ci	***	***	***
WUE	***	***	***
Fo	***	***	***
Fv/Fo	***	***	***
Fv/Fm	***	***	***
qP	ns	ns	ns
SPS	***	***	***
SS	***	***	***
AI	***	***	***
Starch	***	***	*
SP	***	***	*
α-Amylase	***	***	**
β-Amylase	***	*	ns

*p ≤ 0.05; **p ≤ 0.01 ***p ≤ 0.001; ns, not significant.

*As, arsenic; AMF, arbuscular mycorrhizal fungi; Pn, net photosynthetic rate; E, transpiration rate; Gs, stomatal conductance; Ci, intercellular CO_2_ concentration; WUE, water use efficiency; Fo, minimal fluorescence; Fv/Fo, potential efficiency of PSII; Fv/Fm, maximum efficiency of PSII; qP, photochemical quenching coefficient; SPS, sucrose-phosphate synthase; SS, sucrose synthase; AI, acid invertase; SP, starch phosphorylase.*

### Sugar Metabolism

#### Total Soluble Sugar, Reducing Sugar, and Non-reducing Sugar Concentrations

Two-way ANOVA revealed that As level, mycorrhizal status, and interaction of both these factors significantly affected concentrations of TSS and NRS in wheat leaves. Exposure of plants to As increased concentrations of TSS and RS at both As levels and decreased NRS concentration with increased As level in soil. However, mycorrhizal colonization showed a varied effect on sugar concentrations (Table 4). While mycorrhizal colonization significantly (*p* ≤ *0.05*) increased TSS and NRS concentration at 25 and 50As, RS concentration increased significantly (*p* ≤ 0.05) at 50As only. In response to mycorrhizal colonization, wheat plants showed increase in TSS concentration by 41.1 and 35.3% at 25 and 50As, respectively, when compared with NM plants. A similar trend was observed in case of NRS. When compared with NM plants, the concentration of NRS increased in M plants by 106.5 and 117.2% at 25 and 50As, respectively. However, a positive effect of *R. intraradices* colonization on RS concentration was evident only at 50As level, as RS concentration significantly increased by 6.6% in M plants when compared with NM plants exposed to a similar As level.

**TABLE 4:** Concentrations of TSS, RS, NRS, and NRS/RS ratio in leaves of *Triticum aestivum* in response to *Rhizophagus intraradices* inoculation (M, mycorrhizal; NM, non-mycorrhizal) and As addition to the soil.

As level (mg As kg^−1^ soil)	AMF status	TSS (mg g^–1^ FW)	RS (mg g^–1^ FW)	NRS (mg g^–1^ FW)	NRS/RS ratio
0	NM	8.59 ± 0.17d	3.27 ± 0.33d	5.32 ± 0.49b	1.62
	M	9.74 ± 0.77d	4.04 ± 0.55d	5.69 ± 0.41b	1.40
25	NM	13.20 ± 1.12c	8.72 ± 0.17c	4.47 ± 1.12b	0.51
	M	18.62 ± 0.12b	9.39 ± 0.46c	9.23 ± 0.34a	0.98
50	NM	20.54 ± 0.47b	16.13 ± 0.37b	4.41 ± 0.68b	0.27
	M	27.79 ± 2.70a	17.20 ± 0.23a	9.58 ± 1.24a	0.55
Significance As		***	***	*	
AMF		***	***	***	
As × AMF		***	ns	***	

Values represent means of three biological replicates ± SD. Different letters within the columns represent significant difference among the treatments at *p* ≤ 0.05, derived from Tukey HSD.

*** represent significance at *p* ≤ 0.05, *p* ≤ 0.01, and *p* ≤ 0.001, respectively, derived from two-way ANOVA.

*ns, not significant. As, arsenic; AMF, arbuscular mycorrhizal fungus; NRS, non-reducing sugars; RSS, reducing sugar; TSS, total soluble sugars; HSD, honestly significant difference.*

#### Sucrose-Phosphate Synthase Activity

**FIGURE 4:**
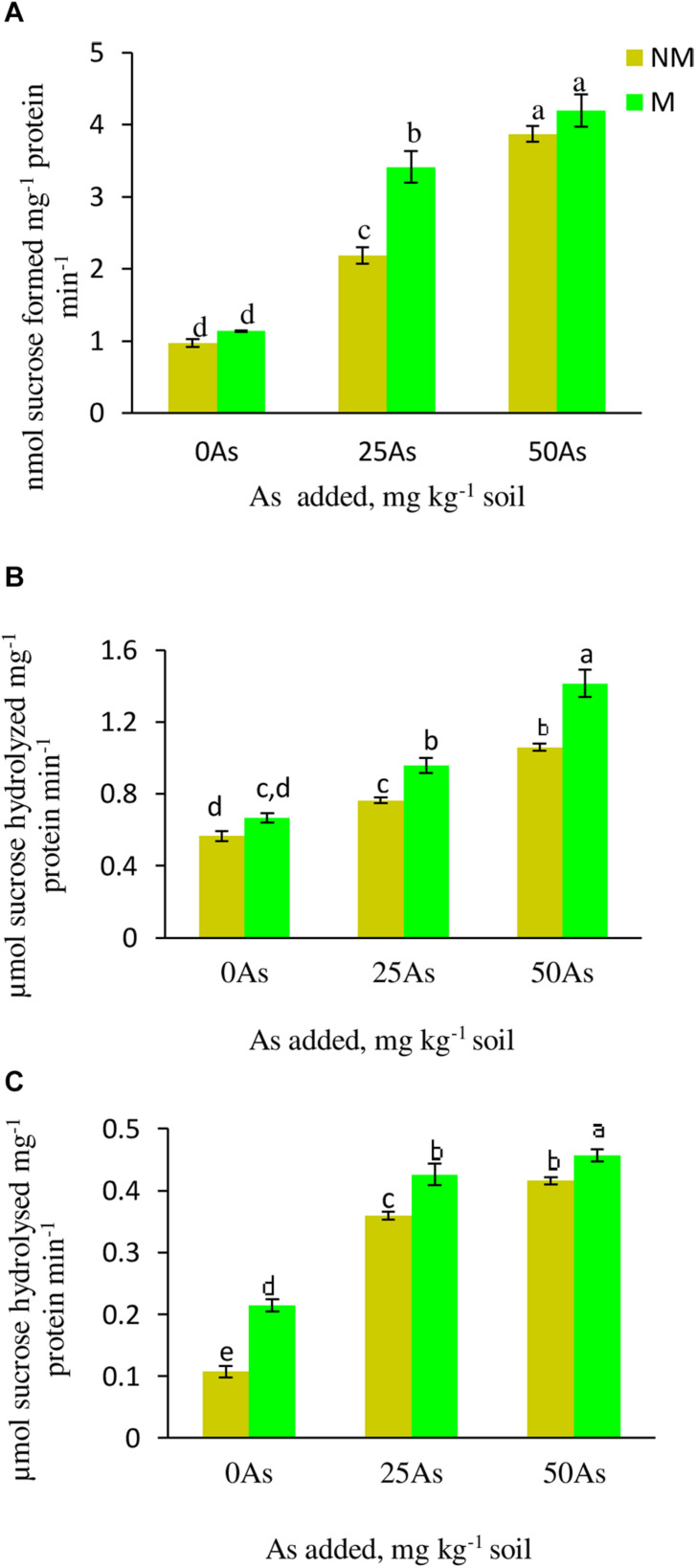
Activity of sucrose metabolizing enzymes **(A)** sucrose phosphate synthase, SPS; **(B)** sucrose synthase, SS; and **(C)** acid invertase, AI, in leaves of *Triticum aestivum* in response to *Rhizophagus intraradices* inoculation (M, mycorrhizal; NM, non-mycorrhizal) and As addition to the soil. Values represent means of three biological replicates ± SD. Different letters represent significant difference at *p* ≤ *0.05*, derived from Tukey’s honestly significant difference (HSD). 0As, 0 mg As kg^–1^ soil treatment; 25As, 25 mg As kg^–1^ soil treatment; 50As, 50 mg As kg^–1^ soil treatment.

With increased concentration of As in soil, the activity of SPS increased significantly (*p* ≤ *0.05*), indicating sensitivity of the enzyme toward As toxicity. When compared with 0As plants, SPS activity increased significantly by 124.7 and 298.9% at 25 and 50As, respectively (Figure 4). Colonization by *R. intraradices* enhanced the enzyme activity by 16.5, 62.4, and 8.3% at 0, 25, and 50As, respectively, in M plants over their corresponding NM plants.

#### Sucrose Synthase and Acid Invertase Activities

Both the activities of SS and AI increased in plants exposed to As in a concentration-dependent manner. At 25As, SS and AI activities of NM plants were enhanced by 35.7 and 25%, respectively, over 0As plants (Figure 4). When compared with 0As level, an increment of 8.9 and 31.0% was observed in SS and AI activities, respectively, in plants grown at 50As. At all As levels, mycorrhizal colonization increased the activities of these enzymes with respect to their NM counterparts. *R. intraradices* colonization increased SS activity by 27.1 and 8.6% and AI activity by 20.0 and 9.7% at 25 and 50As, respectively, over 0As plants.

### Starch Metabolism

#### Starch Concentration

**FIGURE 5:**
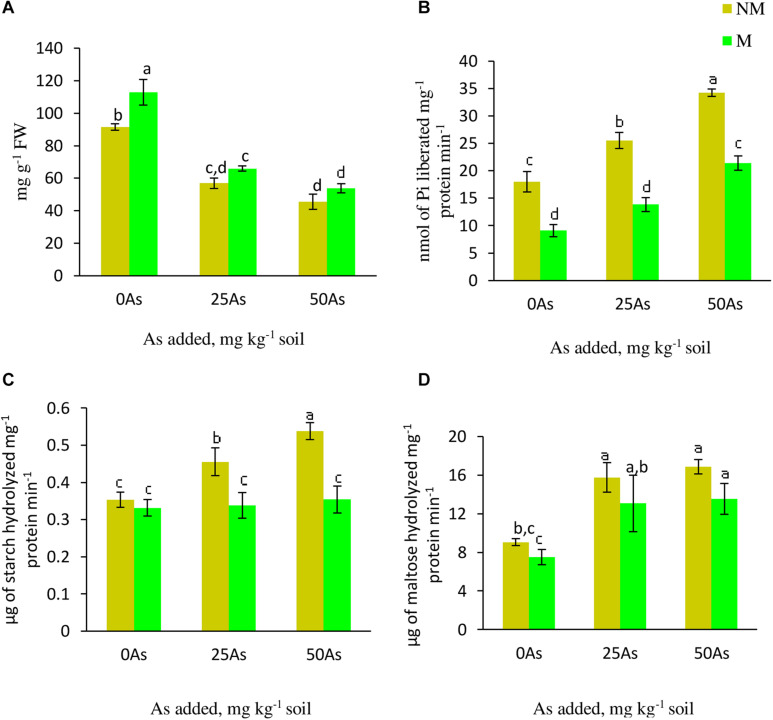
Concentration of **(A)** starch and activities of starch metabolizing enzymes; **(B)** starch phosphorylase, SP; **(C)** α-amylase, and **(D)** β-amylase in leaves of *Triticum aestivum* in response to *Rhizophagus intraradices* inoculation (M, mycorrhizal; NM, non-mycorrhizal) and As addition to the soil. Values represent means of three biological replicates ± SD. Different letters represent significant difference at *p* ≤ *0.05*, derived from Tukey’s honestly significant difference (HSD). 0As, 0 mg As kg^–1^ soil treatment; 25As, 25 mg As kg^–1^ soil treatment; 50As, 50 mg As kg^–1^ soil treatment.

As stress resulted in decrease in starch concentration with the lowest concentration reported at 50As. The same trend of decline in the concentration was observed upon mycorrhizal colonization at 25 and 50As. Nevertheless, in comparison with their respective NM counterparts, M plants showed significant (*p* ≤ *0.05*) increase in starch concentration by 23.1% at 0As. However, this AMF-mediated increase was non-significant at 25 and 50As (Figure 5).

#### α-Amylase and β-Amylase Activities

Activities of α- and β-amylase increased in a dose-dependent manner when exposed to low and high concentrations of As. When compared with 0As plants, an increase of 28.6 and 51.4% was observed on α-amylase activity in leaves of NM plants exposed to 25 and 50As, respectively. No influence of *R. intraradices* on α-amylase activity was reported, as indicated by a non-significant effect of mycorrhizal status on its activity at 0As. However, its activity decreased significantly at 25 and 50As by 26.1 and 35.2%, respectively, in comparison with NM plants (Figure 5). Following a similar trend of α-amylase activity, β-amylase showed increased activity with increased As level. In NM plants, increments of 73.9 and 86.4% were observed over control when plants were exposed to 25 and 50As, respectively. However, the degree of increase in β-amylase activity was less in M plants when compared with NM plant of the same As level.

#### Starch Phosphorylase Activity

Statistically significant (*p* ≤ *0.05*) increments in SP activity was observed when plants were exposed to As. SP activity increased by 41.5 and 89.9% at 25 and 50As, respectively, when compared with 0As plants. Inoculation with *R. intraradices* decreased its activity at all As levels when compared with NM plants; nevertheless, with increase in As concentration, its activity increased (Figure 5).

## Discussion

Presence of As in soil increased *R. intraradices* colonization in wheat roots. Higher colonization of wheat roots by *R. intraradices* in the presence of As suggests the tolerance of *R. intraradices* to As. This observation is in congruence with higher spore density of *R. intraradices* in As-contaminated sites over non-contaminated soil reported by Schneider et al. (2013) and Krishnamoorthy et al. (2015). Higher root colonization may be an adaptive strategy of *R. intraradices* to As in soil.

The findings of the present study showed that colonization of *T. aestivum* with *R. intraradices* alleviates the detrimental effects of As stress on the photosynthetic parameters such as pigment concentrations, Hill reaction activity, leaf gaseous exchange, and Chl *a* fluorescence. The effect of As stress on the above parameters has been studied in several plants, including wheat; however, most of these studies are limited to seedling stage (Chen et al., 2017; Sil et al., 2019; Majumder et al., 2020). While seedlings depend on reserves stored in seed endosperm (Ferreira et al., 2009), mature plants primarily meet their nutritional need through photosynthesis and display a more complex source–sink relationship (Yu et al., 2015). Further, it is known that formation of AM in roots creates a strong carbon sink and thus influence photosynthesis of plants (Gavito et al., 2019). Study on the long-term effect of As on plants and the role of AMF in amelioration of As stress requires assessment on mature plants. To the best of our knowledge, this is the first study to provide evidence for amelioration of As-induced perturbation in carbohydrate metabolism by AMF inoculation.

The physiological and biochemical changes in plants due to As contamination in soil can be attributed to As toxicity, ionic imbalance, and replacement of essential elements with As in various enzymatic reactions (Sharma et al., 2017; Alam et al., 2019). The first and foremost reason for mitigation of As-induced damage in photosynthetic machinery in M plants can be attributed to reduced uptake and translocation of As in leaves of *T. aestivum* observed in the study and improved antioxidant potential (Sharma et al., 2017).

Roots get directly exposed to As present in the soil that inhibits its growth and proliferation, resulting in compromised nutrient uptake (Alam et al., 2019). Being a structural analog of inorganic P, As(V) is transported across plasma membrane through the phosphate transport systems, where it competes and interferes with P uptake and metabolism (Stoeva and Bineva, 2003). This caused an increase in As and a decrease in P concentrations in leaves of wheat grown in As-contaminated soil in the present study. Further, wheat plants when exposed to As in soil showed decline in concentrations of N and Mg in leaves. The negative effects of As on the uptake of these nutrients can be directly linked to reduced root growth due to alteration in morphological and physiological characteristics of roots by As (Khudsar et al., 2000). A lower N concentration under As stress can also be due to the disturbances in activities as well as affinities of key enzymes involved in N uptake and metabolism (Ghosh et al., 2013; Sil et al., 2019). Decreased concentration of As in M plants over NM plants in the present study substantiates the potential of *R. intraradices* in mitigating As accumulation in wheat leaves. AMF compensates for the As-mediated root toxicity in plant by providing another route for nutrient uptake by virtue of its extraradical hyphae (Ezawa et al., 2002; Smith and Read, 2008). The extraradical hyphae of AMF ensure access to larger soil volume for acquisition of nutrients (Smith and Read, 2008), augment N assimilation by influencing enzymes of N metabolism (Govindarajulu et al., 2005; Zhu et al., 2016), mobilize immobile nutrients by lowering the pH of rhizosphere (Smith and Read, 2008), and also rapidly transfer Pi as polyphosphate (Ezawa et al., 2002). However, there is no report on transport of As(V) as polyarsenate by an AMF hypha. There are reports that deprivation in concentrations of these nutrients causes closure of reaction centers of PSII, disrupts electron transport chain, and compromises synthesis of photosynthetic pigment that ultimately limits photosynthesis (Singh et al., 2017). An augmentation in concentrations of these nutrients by AMF in present study supports its ameliorative role under As stress.

One of the most consequential responses of plants to As stress is the decline in concentrations of photosynthetic pigments (Gusman et al., 2013; Emamverdian et al., 2015). Concentrations of Chl *a*, Chl *b*, and total carotenoids decreased in NM as well as M plants when subjected to As stress. Concentrations of all pigments were significantly higher in M plants than NM plants at each level of As contamination in soil. Increase in concentrations of Chl *a* and Chl *b* in M over NM plants has been reported in citrus, cucumber, and chickpea (Li et al., 2013; Chen et al., 2017; Garg and Cheema, 2020). Chl contains tetrapyrrole with Mg in the center and proteins (Fiedor et al., 2008). The decrease in Chl concentrations due to As stress may be due to decrease in the protein, N, and Mg concentrations observed in the present study. The concentration of total proteins was higher in M plants compared with NM plants at all levels of As including control (0As), and this may be attributed to improved N uptake in M plants over NM plants. Kitajima and Hogan (2003) proposed that adjustment of Chl *a*/*b* ratio is an integral feature of acclimatization to low N availability. The Chl *a*/*b* ratio is expected to increase with decline in N concentrations. Carotenoids are accessory pigments that protect Chl *a* and Chl *b* from oxidative damage (Hou et al., 2007). Several studies have related decline in Chl pigments to reduced level of carotenoids (Zhou et al., 2018; Sil et al., 2019; Majumder et al., 2020). Higher concentration of total carotenoids in M than NM plants at all levels of As stress suggests protection of Chl from As-induced oxidative damage.

Among all the photosynthetic pigments, Chl *b* concentration was most affected under As stress, followed by Chl *a* and total carotenoids. An enhancement in Chl *a*/*b* ratio in present study indicates higher degradation of Chl *b* over Chl *a* in the presence of As. There are reports by Sil et al. (2019) and Majumder et al. (2020) stating higher sensitivity of Chl *b* over Chl *a* under As stress. However, the exact mechanism for such differential behavior under As stress needs further investigation. Higher Chl *a*/*b* ratio indicates higher distress on thylakoids (Zhou et al., 2018), which reflects a plant’s inadequacy to transfer electron and excitation energy to the PSII core complex (Xu et al., 1995). Dose-dependent increase in Chl *a*/*b* ratio in *T. aestivum* in present study is in line with the reports on maize, wheat, and rice (Dresler et al., 2014; Sil et al., 2019; Majumder et al., 2020). The increase in Chl *a*/*b* ratio can be ascribed to As-mediated decrease in photosynthetic pigments and Hill reaction activity (Allakhverdiev et al., 2000; Sil et al., 2019). Low Hill reaction activity in plants results in decline in NADP reduction, phosphorylation inactivation, and CO_2_ assimilation (Yang et al., 2009). The decline in CO_2_ assimilation is corroborated in terms of decline in Pn and starch concentration in As-stressed plants observed in this study.

The lower Chl *a*/*b* ratio in M plants over NM plants reflects higher efficiency to transfer excitation energy to the PSII core complex, consequently resulting in more CO_2_ assimilation, higher Pn, and augmented starch concentration in M plants when compared with NM plants. These results show that M plants are superior to NM plants in counterbalancing As-mediated limitation in photosynthetic pigment and Hill reaction activity.

It is well documented that heavy metals affect gas exchange parameters in plants (Tian et al., 2014; Majumder et al., 2020). Contamination of As in soil resulted in reduced Pn and Gs in present study. Normally, RuBisCo (ribulose-1,5-bis-phosphate carboxylase/oxygenase) activity positively affects assimilation of CO_2_ in plants. The decline in Pn under As stress has been ascribed to inactivation of RuBisCo involved in carbon fixation (Finnegan and Chen, 2012). Additionally, As-mediated P deficiency in present study is also a possible reason for lower Pn under As stress. P is involved in synthesis of ATP and other phosphorylated metabolites of photosynthesis. Deficiency of P has been reported to cause closure of PSII reaction centers and to inhibit transfer of electrons from PSII to PSI (Singh et al., 2017). AMF colonization promotes photosynthesis by increasing RuBisCo carboxylation and RuBP (ribulose-1,5-bis-phosphate) regeneration (Chen et al., 2017), and augmenting P uptake. Rai et al. (2014) showed that the presence of As reduces CO_2_ assimilation and subsequently decreases CO_2_ demand that accounts for decline in Gs observed in present study.

Arbuscular mycorrhizal fungi are obligate symbionts that obtain all the carbon needed for their growth and activities from the host plant. Approximately, one-fifth of the carbon fixed as photosynthates is used in sustenance of AM symbiosis (Bago et al., 2000). This increase in carbon demand in combination with higher concentration of photosynthetic pigments, lower Chl a/*b* ratio, and enhanced Hill reaction activity triggers Pn in M plants, leading to higher stomatal conductance and consequently increasing E and Ci.

Among the two energy harvesting centers in plants, PSII is more sensitive to stress than PSI (Björkman and Demmig, 1987; Stoeva and Bineva, 2003; Iriel et al., 2015). PSII photochemistry is represented by Chl *a* fluorescence attributes, namely, Fo, Fv/Fo, and Fv/Fm (Zhu et al., 2014). Decline in these features due to As in *T. aestivum* indicates compromised functionality of PSII, damage to photosynthetic apparatus, and photoinhibition (Stoeva and Bineva, 2003; Wang et al., 2016). Depreciation in PSII efficiency due to As stress has also been reported in *Oryza sativa* and *Glycine max* (de Andrade et al., 2015; Piršelová et al., 2016). Nevertheless, colonization of wheat plants by *R. intraradices* protected PSII reaction center from As-mediated damage. Under As stress, M plants showed lesser decrease in Fv/Fm than did NM plants. It can be inferred that adverse effects of As on photochemistry of PSII of wheat plants can be alleviated by inoculation with *R. intraradices*, which aid in improving As tolerance.

Sugars generated by photosynthesis, besides serving as substrates in cellular respiration that fuel metabolism, also play a pivotal role in the maintenance of growth, osmotic homeostasis, and membrane stabilization of plant cells (Muller et al., 2011; Bouthour et al., 2012; Sami et al., 2016). On the other hand, starch is the main storage carbohydrate in plants. Under stressful conditions, breakdown of starch results in accumulation of soluble sugars to carry out basal metabolism to sustain plant’s growth and development (Stitt and Zeeman, 2012; Begum et al., 2019; Gupta and Thind, 2019). The increment in concentration of TSS and decline in starch concentration with increase in As stress in the present study is in agreement with the above statements. This is further supported by increase in the activities of the starch-degrading enzymes.

Enhanced concentration of TSS generates a feedback inhibition of Pn (Goldschmidt and Huber, 1992). The limitation in Pn activates remobilization of starch as observed in the present study, as indicated by enhanced activities of starch-hydrolyzing enzymes. Additionally, decline in starch concentration can also be credited to As-mediated restrain on starch synthesizing enzymes as reported by Yadav (2010). Findings of the study indicate that when subjected to As-treated soil, plants sustain basic metabolism by enhancing sugars accumulation, limiting Pn, and augmenting starch degradation. Contrary to this, inoculation of wheat plants with *R. intraradices* mitigated effects of As on starch-hydrolyzing enzymes, as indicated by enhanced starch concentration as a consequence of decreased activities of starch-degrading enzymes. This can be explained by (i) lower requirement of sugar due to lower intensity of As stress (low As concentration) in M plants and (ii) higher Pn that contributes to sugar requirement under As stress and further reduces starch degradation.

The increase in RS and NRS together contributed to increase in concentrations of TSS in NM and M plants. The increase in TSS and RS concentrations in response to As can be linked to increment in activities of sucrose-metabolizing enzymes and breakdown of starch as indicated by elevated activities of starch-degrading enzymes in the present study. Sucrose-synthesizing enzyme, SPS, is reported to be influenced by abiotic as well as biotic stress conditions (Krause et al., 1998). Increase in activity of SPS at 25 and 50As was also observed in wheat plants under As stress. Soon after its synthesis, sucrose is degraded by sucrose-metabolizing enzymes to produce RS as observed in this study as well. The observed changes are consistent with the report of Choudhury et al. (2010). Increase in AI activity under heavy metal stress facilitates production of hexoses that aid in quenching free radicals and also ensure instigation of ROS metabolism via oxidative pentose phosphate pathway (Peshev et al., 2013). Interestingly, while in the absence of As, the ratio of NRS/RS was higher in NM plants; under increasing As stress, NRS/RS ratio was higher in M plants. This implies that under As stress, RS was synthesized more than NRS in NM plants, and NRS contributed more to the increase in concentration of TSS in M plants. The variations in relative proportion of sugars between NM and M plants can be explained on the basis that while NM plants require RS to scavenge As-induced ROS, M plants are already endowed with high enzymatic and non-enzymatic antioxidants (Sharma et al., 2017). On the other hand, M plants require higher concentration of sucrose (NRS) for long-distance transport to meet the high demand of sugars to maintain AM symbiosis (Ward et al., 1997).

## Conclusion

As-mediated perturbations in wheat plants resulted in a dose-dependent decline in gas exchange parameters, namely, Pn, Gs, Ci, and E. Apparent decrease in pigment concentration along with Hill reaction activity was also reported under As stress. Several factors contribute to ameliorative effects of AM on photosynthesis such as higher concentration of photosynthetic pigments, favorable Chl *a*/*b* ratio, higher Hill reaction activity, and PSII efficiency that ultimately depend upon As concentration in the leaf tissue and uptake of mineral nutrients such as P, N, and Mg. Additionally, increased carbon demand as a result of formation of AM prevents feedback inhibition of photosynthesis due to As-induced reduction of CO_2_ assimilation. Higher Pn in M plants reduced the need for starch degradation to form sugars. Furthermore, proportion of NRS (sucrose) was higher in M plants that endowed better ability to tolerate As stress. Therefore, deployment of AMF as a biofertilizer in As-contaminated regions to refurbish physiological as well as biochemical impediments in wheat for improved growth is highly suggested.

## Data Availability Statement

The original contributions presented in the study are included in the article/supplementary material, further inquiries can be directed to the corresponding author.

## Author Contributions

RK designed and planned the experiment. SG executed the experiments and analyzed the results. RK, SG, and ST jointly wrote the manuscript. All the authors have collectively reviewed the manuscript and approved it.

## Conflict of Interest

The authors declare that the research was conducted in the absence of any commercial or financial relationships that could be construed as a potential conflict of interest.

## Abbreviations

AI: acid invertase
AMF: arbuscular mycorrhizal fungi
ANOVA: analysis of variance
As(III): arsenite
As(V): arsenate
As: arsenic
CaCl_2_: calcium chloride
Chl: chlorophyll
C_*i*_: intercellular CO_2_ concentration
DCPIP: 2,6-dichlorophenolindophenol
DNSA: 3,5-dinitrosalicylic acid
DTT: dithiothreitol
E: transpiration rate
EDTA: ethylenediaminetetraacetic acid
Fo: minimal fluorescence
Fv/Fo: potential of PSII
Gs: stomatal conductance
HSD: honestly significant difference
INVAM: International Culture Collection of (Vesicular) Arbuscular Mycorrhizal Fungi
M: mycorrhizal
MgCl_2_: magnesium chloride
MTI: metal tolerance index
NM: non-mycorrhizal
NRS: non-reducing sugar
Pi: inorganic phosphate
PMSF: phenylmethylsulfonyl fluoride
Pn: net photosynthetic rate
PS: photosystem
qP: quenching coefficient
ROS: reactive oxygen species
RS: reducing sugar
SP: starch phosphorylase
SPS: sucrose-phosphate synthase
SPSS: Statistical Package for the Social Sciences
SS: sucrose synthase
TCA: trichloroacetic acid
T-Chl: total chlorophyll
TSS: total soluble sugar.
